# Cdk5 regulatory subunit-associated protein 1 knockout mice show hearing loss phenotypically similar to age-related hearing loss

**DOI:** 10.1186/s13041-021-00791-w

**Published:** 2021-05-17

**Authors:** Toru Miwa, Fan-yan Wei, Kazuhito Tomizawa

**Affiliations:** 1 Department of Otolaryngology-Head and Neck Surgery, Graduate School of Medicine, Kyoto University, 54 Shogoin Kawahara-cho, Sakyo-ku, Kyoto, 6068507 Japan; 2 Department of Otolaryngology-Head and Neck Surgery, Kitano Hospital, Tazuke Kofukai Medical Research Institute, 2-4-20 Ougimaci, Kita-ku, Osaka, 5308480 Japan; 3 Department of Molecular Physiology, Faculty of Life Sciences, Kumamoto University, 1-1-1- Honjo, Chuo-ku, Kumamoto, 8608556 Japan; 4 Department of Modomics Biology and Medicine, Institute of Development, Aging and Cancer, Tohoku University, 4-1 Seiryo, Aoba-ku, Sendai, Miyagi 9808575 Japan

**Keywords:** Auditory brainstem responses, Cdk5rap1, Endocochlear potentials, Hearing loss, Mitochondrial tRNA, Spiral ligament of cochlea

## Abstract

Mitochondrial dysfunction is associated with aging and age-related hearing loss (AHL). However, the precise mechanisms underlying the pathophysiology of hearing loss remain unclear. Cdk5 regulatory subunit-associated protein 1 (CDK5RAP1) enables efficient intramitochondrial translation by catalyzing the deposition of 2-methylthio modifications on mitochondrial tRNAs. Here we investigated the effect of defective mitochondrial protein translation on hearing and AHL in a *Cdk5rap1* deficiency C57BL/6 mouse model. Compared to control C57BL/6 mice, *Cdk5rap1*-knockout female mice displayed hearing loss phenotypically similar to AHL from an early age. The premature hearing loss in *Cdk5rap1*-knockout mice was associated with the degeneration of the spiral ligament and reduction of endocochlear potentials following the loss of auditory sensory cells. Furthermore, cultured primary mouse embryonic fibroblasts displayed early onset of cellular senescence associated with high oxidative stress and cell death. These results indicate that the CDK5RAP1 deficiency-induced defective mitochondrial translation might cause early hearing loss through the induction of cellular senescence and cochlear dysfunction in the inner ear. Our results suggest that the accumulation of dysfunctional mitochondria might promote AHL progression. Furthermore, our findings suggest that mitochondrial dysfunction and dysregulated mitochondrial tRNA modifications mechanistically cause AHL. Understanding the mechanisms underlying AHL will guide future clinical investigations and interventions in the attempt to mitigate the consequences of AHL.

**Supplementary Information:**

The online version contains supplementary material available at 10.1186/s13041-021-00791-w.

## Introduction

**Fig. 1:**
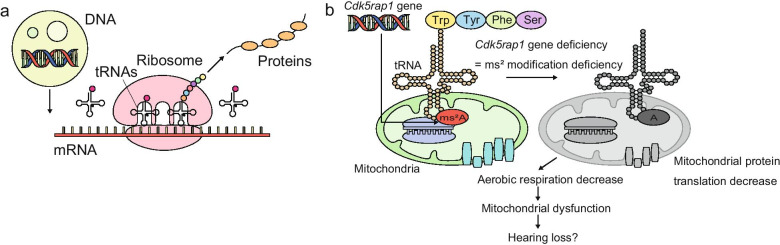
Cdk5 regulatory subunit-associated protein 1 (CDK5RAP1) mediates 2-methylthio (ms^2^) modifications in mammalian mitochondrial transfer RNAs.** a** Central dogma: mt-tRNAs play a crucial role in mitochondrial protein synthesis. **b** Structures of N^6^-isopentenyladenosine (A) and 2-methylthio-N^6^-isopentenyladenosine (ms^2^A)

Age-related hearing loss (AHL) exhibits a multifactorial underlying mechanism. Previous studies suggested that accumulation of damaged mitochondrial DNA (mtDNA), mitochondrial redox imbalance, production of reactive oxygen species (ROS), and compromised antioxidant capacity caused by aging-related oxidative stress are associated with cochlear senescence and AHL in humans and mice [1–5]. While regulating cell signaling and programmed cell death, mitochondria are essential for energy supply and cellular redox homeostasis maintenance [6]. Reportedly, the translation accuracy of the mitochondrial ribosomes impacts cytoplasmic proteostasis and nuclear gene expression, which underlie aging [7–10]. The nucleotides of transfer RNAs (tRNAs) are often post-transcriptionally modified by various enzymatic reactions [11] that are critical for efficient and accurate decoding, especially as they improve tRNA–codon binding [12, 13]. Numerous human diseases such as type 2 diabetes and mitochondrial diseases have been linked to deficient modifications of the mitochondrial tRNAs (mt-tRNAs) [9–11, 14–20]. Dysregulation of tRNA modifications is also thought to precede aging in human [9, 10]. Furthermore, mammalian mt-tRNAs are modified by nuclear tRNA-modifying enzymes such as Cdk5 regulatory subunit-associated protein 1 (CDK5RAP1), which catalyzes the deposition of the 2-methylthio (ms^2^) modifications on the mammalian mt-tRNAs [16, 21] (Fig. 1). Deficient ms^2^ modifications markedly impair mitochondrial protein synthesis under stress conditions, resulting in respiratory defects such as those observed in *Cdk5rap1*-knockout (KO) mice, which are susceptible to stress-induced mitochondrial remodeling [22]. However, the importance of ms^2^ modifications in AHL in mammals remains unknown. CDK5RAP1 may contribute to mitochondrial function of the inner ear cells under stress conditions such as aging-related oxidative stress accumulation by catalyzing the ms^2^ modifications of mt-tRNAs [11, 14, 23]. In this study, we validated this hypothesis by investigating the morphological and functional changes in the inner ear upon aging in the *Cdk5rap1*-KO mice which are deficient in ms^2^ mt-tRNA modifications.

## Results

### Hearing loss occurs at a young age in Cdk5rap1-KO mice

**Fig. 2:**
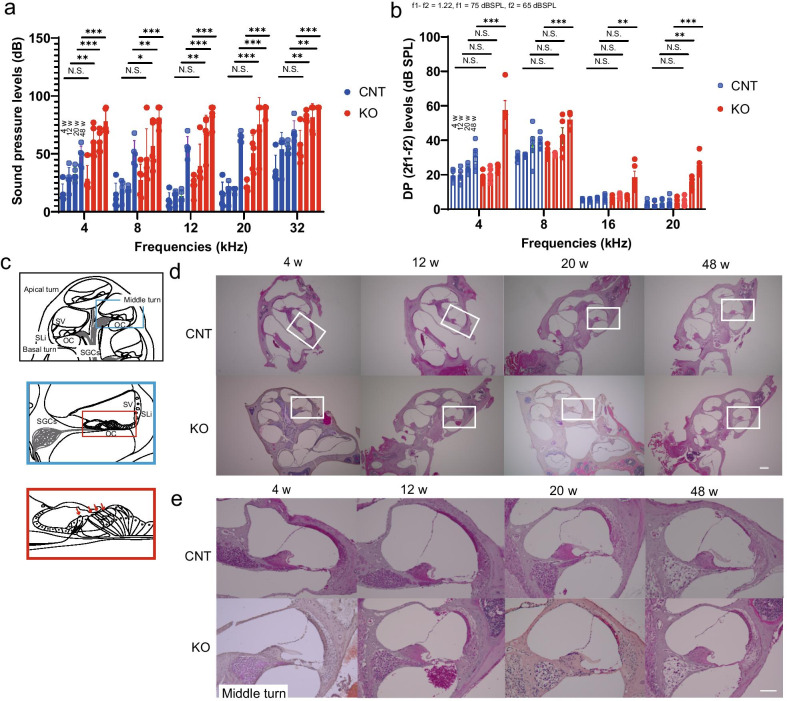
Mitochondrial stress accelerates hearing loss in *Cdk5rap1*-KO mice.** a** Auditory brainstem response thresholds in *Cdk5rap1*-knockout (KO) mice at 4, 12, 20, or 32 weeks of age compared to those in littermate control (CNT) mice at different frequencies (*n* = 5 for each genotype). Error bars represent the mean ± SE of five independent experiments analyzed using the Tukey test. *P < 0.05, **P < 0.01, ***P < 0.001. N.S. not significant. **b** Distortion-product otoacoustic emissions threshold at different frequencies in *Cdk5rap1*-KO mice at 4, 12, 20, or 32 weeks of age compared to those in CNT mice (*n* = 5 for each genotype). Error bars represent the mean ± SE of five independent experiments analyzed using the Tukey test. *P < 0.05, **P < 0.01, ***P < 0.001. N.S. not significant. **c** Illustration of a cross-section through a mouse cochlea cut along its longitudinal axis. *SGCs* spiral-ganglion cells, *OC* organ of Corti, *SV* stria vascularis, *SLi* spiral ligament. The blue and red boxes represent a single half-turn of the cochlea and the zoomed-in view of the OC, respectively. Red arrows indicate specific cell types: inner and outer hair cells. The inner and outer hair cells act as mechanoelectrical transducers and play a crucial role in hearing. The electrical signal is transmitted via the SGCs to the auditory pathway of the brain. **d** Gross morphologies of KO or CNT cochleae (*n* = 5 for each genotype). White boxes indicate the cochlear middle turn. **e** Morphology of the cochlear middle turn in KO or CNT mice at 4, 12, 20, or 48 weeks (*n* = 5 for each genotype). Scale bar = 100 µm

The auditory brainstem response (ABR) is a neural reaction to sonic waves and is used to assess the auditory responses in humans and test animals. The ABR thresholds of 4-week-old *Cdk5rap1-*KO mice did not differ significantly from those of the littermate control (CNT) mice across all tested frequencies (Fig. 2a and Additional file 1: 4 kHz, *P* = 0.09; 8 kHz, *P* = 0.12; 12 kHz, *P* = 0.20; 20 kHz, *P* = 0.21; 32 kHz, *P* = 0.05; analysis of variance (ANOVA) and post hoc Tukey test, F(9,30) = 12.48, *P* < 0.001). However, the ABR thresholds at all frequencies for the 12-, 20-, and 48-week-old KO mice were significantly higher than those of the same age CNT mice (Fig. 2a and Additional file 1: 12 weeks: 4 kHz, *P* = 0.001; 8 kHz; *P* = 0.02; 12 kHz, *P* = 0.009; 20 kHz, *P* = 0.001; 32 kHz, *P* = 0.001; 20 weeks: 4 kHz, *P* < 0.001; 8 kHz, *P* = 0.004; 12 kHz, *P* < 0.001; 20 kHz, *P* < 0.001; 32 kHz, *P* = 0.002; 48 weeks: 4 kHz: *P* < 0.001; 8 kHz, *P* < 0.001; 12 kHz, *P* < 0.001; 20 kHz, *P* < 0.001; 32 kHz, *P* < 0.001; ANOVA and post hoc Tukey test). Furthermore, the distortion-product otoacoustic emissions (DPOAE) of the 4, 12, and 20-week-old *Cdk5rap1-*KO mice did not differ significantly from those of the CNT mice across all tested frequencies except for 20 kHz at 4 and 20 weeks of age (Fig. 2b: 4 weeks: 4 kHz, *P* = 0.99; 8 kHz, *P* = 0.96; 16 kHz, *P* = 0.99; 20 kHz, *P* < 0.001; 12 weeks: 4 kHz, *P* = 0.99; 8 kHz; *P* = 0.99; 16 kHz, *P* = 0.99; 20 kHz, *P* = 0.99; 20 weeks: 4 kHz, *P* = 0.99; 8 kHz, *P* = 0.67; 16 kHz, *P* = 0.99; 20 kHz, *P* = 0.006; ANOVA and post hoc Tukey test, F(21,128) = 4.41, *P* < 0.001). However, the DPOAE of the KO mice at 48 weeks of age were significantly higher than those of the CNT mice at all frequencies (Fig. 2b: 48 weeks: 4 kHz: *P* < 0.001; 8 kHz, *P* < 0.001; 16 kHz, *P* = 0.003; 20 kHz, *P* < 0.001; ANOVA and post hoc Tukey test).

### Cochlear morphology

For morphological assessment, we performed H&E staining. Gross cochlear morphology of the *Cdk5rap1-*KO and CNT mice appeared normal. However, loss of the spiral-ganglion cells (SGCs) was observed in the cochleae of KO mice at 12, 20, and 48 weeks of age as well as in the cochleae of CNT mice at 20 and 48 weeks of age (Fig. 2c, d and Additional file 2a, b). In addition, a loss of fibrocytes in the spiral ligament (SLi) was observed in the cochleae of the 4-, 12-, 20-, and 48-week-old KO mice and in those of the 20- and 48-week-old CNT mice (Fig. 2c–e and Additional file 2c). Nevertheless, stria vascularis (SV) from the KO mice did not differ morphologically from that of the CNT mice at any age.

### Early loss of HCs and SGCs in KO mice

**Fig. 3:**
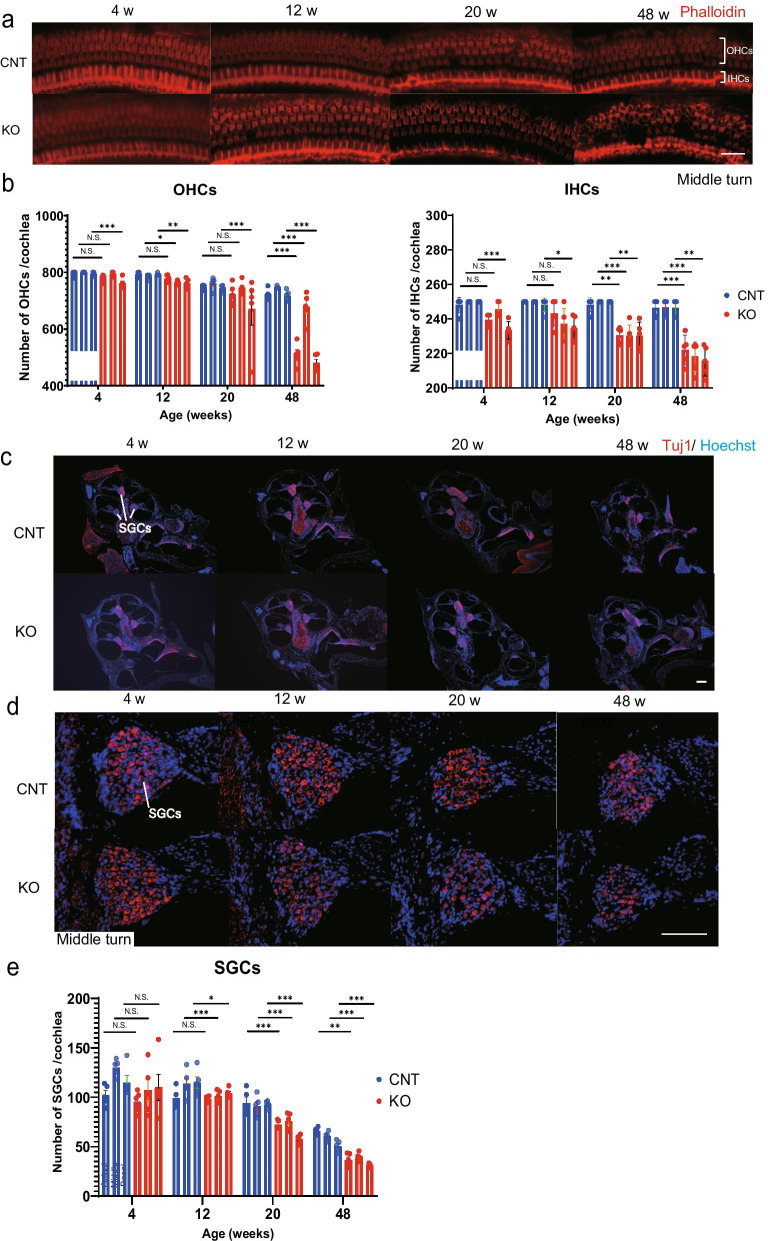
Mitochondrial stress accelerates the loss of HCs and SGCs in *Cdk5rap1*-KO mice. **a**, **b** Changes in the number of IHCs and OHCs in *Cdk5rap1*-knockout (KO) and littermate control (CNT) cochleae with age (*n* = 5 for each genotype). **c–e** Changes in SGC counts in KO and CNT mice with age (*n* = 5 for each genotype). *SGCs* spiral-ganglion cells, *OHCs* outer hair cells, *IHCs* inner hair cells. Scale bars = 100 µm

To examine the effect of *Cdk5rap1-*KO on the auditory sensory cells of aging mice, we immunostained and counted hair cells (HCs) and SGCs. The number of the outer hair cells (OHCs) and the inner hair cells (IHCs) of both KO and CNT mice gradually decreased with age (Fig. 3a, b). At 4 weeks of age, the number of OHCs did not significantly differ between the KO and CNT mice, except at the basal turn (Fig. 3a and b: apical, *P* = 0.12; middle, *P* = 0.55; basal, *P* < 0.001; F(8, 15) = 10.4, *P* < 0.001). However, the number of OHCs observed in the 12-week-old KO mice was significantly lower than that in their age-matched CNT counterparts, except at the apical turn (Fig. 3a, b: apical, *P* = 0.55; middle, *P* = 0.01; basal, *P* < 0.001). The OHC number in 20-week-old KO mice was not significantly lower than that in the CNT mice of a similar age, except at the basal turn (Fig. 3a, b: apical, *P* = 0.09; middle, *P* = 0.24; basal, *P* < 0.001). Alternatively, the OHC number in 48-week-old KO mice was significantly lower than that in their age-matched CNT counterparts (Fig. 3a and b: apical, *P* < 0.001; middle, *P* < 0.001; basal, *P* < 0.001). At 4 and 12 weeks of age, the number of IHCs did not significantly differ between *Cdk5rap1-*KO and CNT, except at the basal turn (Fig. 3a, b: 4 weeks: apical, *P* = 0.14; middle, *P* = 0.12; basal, *P* = 0.001; 20 weeks: apical, *P* = 0.12; middle, *P* = 0.12; basal, *P* = 0.01; F(8, 15) = 56.6, *P* < 0.001). However, the number of IHCs in the KO mice at 20 and 48 weeks of age was significantly lower than that in their age-matched CNT counterparts (Fig. 3a, b: 20 weeks: apical, *P* = 0.01; middle, *P* = 0.003; basal, *P* < 0.001; 48 weeks: apical, *P* < 0.001; middle, *P* < 0.001; basal, *P* = 0.001). Lastly, the number of SGCs in both KO and CNT mice decreased gradually in an age-dependent manner (Fig. 3c, d). At 4 weeks of age, the number of SGCs did not significantly differ between *Cdk5rap1* -KO and CNT mice (Fig. 3c, d: apical, *P* = 0.15; middle, *P* = 0.20; basal, *P* = 0.97; F(8, 15) = 1.26, *P* = 0.03). However, at 12 weeks of age, a significant difference was observed, except at the apical turn (Fig. 3d: apical, *P* = 0.16; middle, *P* < 0.001; basal, *P* = 0.02). Furthermore, the number of SGCs in the 20- and 48-week-old KO mice was significantly lower than that in the CNT mice (Fig. 3c, d: 20 and 48 weeks: all turns, *P* < 0.001). Altogether, these results indicate that the number of OHCs, IHCs, and SGCs decreased earlier in the *Cdk5rap1-*KO mice than in the CNT mice.

### Senescence in SGCs and SV of KO mice

**Fig. 4:**
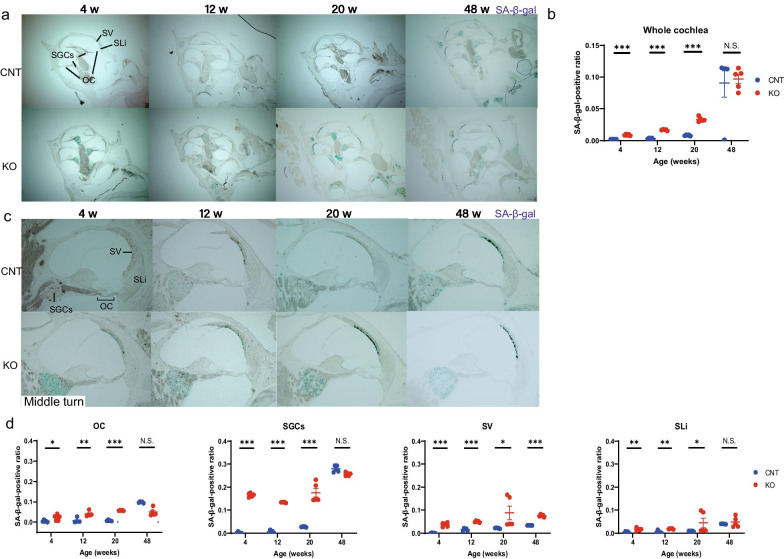
Cochlear senescence in SGCs and stria vascularis (SV) cells of *Cdk5rap1*-KO mice. **a** Sections of cochlea stained with senescence-associated β-gal (SA-β-gal) in *Cdk5rap1*-knockout (KO) and littermate control (CNT) mice at 4, 12, 20, or 48 weeks of age. Senescence-positive cells appear blue. *SGCs* spiral-ganglion cells, *OC* organ of Corti, *SV* stria vascularis, *SLi* spiral ligament. Scale bar = 100 µm. **b** SA-β-gal-positive ratio in the cochleae of KO or CNT mice (*n* = 5 for each genotype). **c** Magnified image of the middle cochlear turn illustrating the distribution of senescent cells. Scale bar = 100 µm. **d** SA-β-gal-positive ratio in the separate areas of KO and CNT cochlea (*n* = 5 for each genotype)

We measured the SA-β-gal activity to assess cellular aging [24, 25] in the cochlea of *Cdk5rap1-*KO mice. Senescent cells were detected in the organ of Corti (OC) and the SLi at 4, 12, 20, and 48 weeks of age in the cochleae of *Cdk5rap1-*KO mice and at 48 weeks in the cochleae of CNT mice (Fig. 4a–d and Additional file 3a, c). Senescent cells were detected among SGCs and cells in the SV at 4, 12, 20, and 48 weeks of age in the cochleae of *Cdk5rap1-*KO mice and 12, 20, as well as at 48 weeks of age in the cochleae of CNT mice (Fig. 4a–d and Additional file 3b). Senescent marginal cells in the SV of the 4 week-old cochleae of *Cdk5rap1-*KO mice gradually became more widespread and were observed in the marginal as well as the intermediate cell populations at 20 weeks of age (Fig. 4c and Additional file 3c). Senescent marginal, intermediate, and basal cells were detected in both KO and CNT SVs at 48 weeks of age. However, the number of the senescent cells in the cochleae of *Cdk5rap1-*KO mice was higher than that observed in the cochleae of CNT mice (Fig. 4d). A different senescence marker, lipofuscin, was observed using H&E staining in approximately the same areas as the SA-β-gal activity in the cochlea (Additional file 4a–c). The cochlear SA-β-gal-positive ratio was higher in the KO animals than that in the CNT animals (Fig. 4b: 4 weeks: 0.008 vs. 0.002, *P* < 0.001, 12 weeks: 0.016 vs. 0.002, *P* < 0.001, 20 weeks: 0.03 vs. 0.008, *P* < 0.001; 48 weeks: 0.096 vs. 0.091, *P* = 0.81; F(1, 5) = 13.6, *P* = 0.01). Lastly, this ratio was higher in the cochlear OC, SGCs, SV, and SLi of the KO animals than those observed in the CNT animals (Fig. 4d: OC: 4 weeks, 0.02 vs. 0.004, *P* = 0.04; 12 weeks, 0.04 vs. 0.007, *P* = 0.002; 20 weeks, 0.05 vs. 0.007, *P* < 0.001; 48 weeks, 0.097 vs. 0.098, *P* = 0.94; F(1, 5) = 13.4, *P* = 0.01, SGCs: 4 weeks, 0.16 vs. 0.002, *P* < 0.001; 12 weeks, 0.13 vs. 0.005, *P* < 0.001; 20 weeks, 0.17 vs. 0.02, *P* = 0.001; 48 weeks, 0.25 vs. 0.27, *P* = 0.05; F(1, 5) = 6.03, *P* = 0.04; SV: 4 weeks, 0.03 vs. 0.0007, *P* < 0.001; 12 weeks, 0.05 vs. 0.01, *P* < 0.001; 20 weeks, 0.69 vs. 0.02, *P* = 0.02; 48 weeks, 0.07 vs. 0.03, *P* < 0.001; F(1, 5) = 100.0, *P* < 0.001; SLi: 4 weeks, 0.01 vs. 0.003, *P* = 0.006; 12 weeks, 0.01 vs. 0.005, *P* = 0.003; 20 weeks, 0.04 vs. 0.009, *P* = 0.01; 48 weeks, 0.048 vs. 0.041, *P* = 0.47; F(1, 5) = 21.3, *P* = 0.005).

### Mitochondrial metabolites accumulate in the KO cochleae

**Fig. 5:**
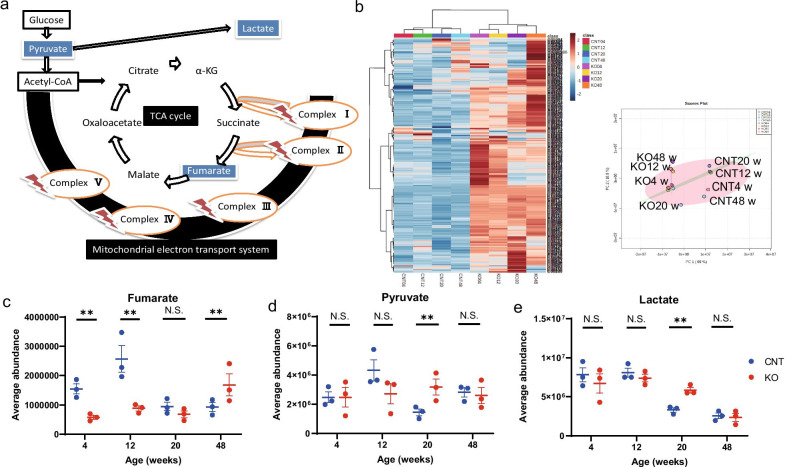
Changes in mitochondrial metabolites in *Cdk5rap1*-KO cochleae. **a** Schematic representation of the tricarboxylic acid (TCA) cycle and mitochondrial electron-transport system. Under stress conditions, the mitochondrial respiratory chain complexes’ activities decreased in *Cdk5rap1*-knockout (KO) mice. **b** Heatmap analysis (left panel) and principal-component analysis (right panel) as components of metabolome analysis; *n* = 3 for each genotype **(c).** Comparison of fumarate, pyruvate, and lactate levels between the cochleae of KO and littermate control (CNT) mice at 4, 12, 20, or 48 weeks of age; *n* = 3 for each genotype

To verify whether aging-associated stress leads to a decrease in the mitochondrial oxidative metabolite levels through a deficient ms^2^ modification of mt-tRNAs, we performed cochlear metabolomic analyses at different time points. Heatmap and principal-component analyses revealed metabolite differences between the CNT and *Cdk5rap1-*KO cochleae at every stage (Additional files 5, 6). The metabolic pathways enhanced in the KO cochleae differed from those enhanced in the CNT cochleae at different ages. In 4-week-old animals, these pathways were associated with the tricarboxylic acid (TCA) cycle, transfer of acetyl groups into mitochondria, and mitochondrial β-oxidation of long-chain fatty acids (Additional file 7). Furthermore, β-alanine metabolism, threonine, and 2-oxobutanoate degradation, vitamin K metabolism, and β-oxidation of very-long-chain fatty acids pathways were observed to be influenced in 12-week-old animals. In 20-week-old animals, steroidogenesis, starch and sucrose metabolism, as well as the mitochondrial electron-transport chain pathways were affected. At 48 weeks of age, the phospholipid biosynthesis, the Warburg effect, and tyrosine and pyruvate metabolism pathways were observed to be influenced (Additional file 7). Furthermore, we observed significant changes in the levels of fumarate, pyruvate, and lactate, which are metabolites related to the TCA cycle (Fig. 5a). Figure 5b illustrates the heatmap and principal-component analyses for the data generated during metabolome analysis at each stage. Mitochondrial dysfunction was previously shown to be consistent with the decrease in fumarate levels and increase in pyruvate and lactate levels [26, 27]. We observed a significant reduction in fumarate concentration in the cochlea of *Cdk5rap1-*KO mice compared to that of the CNT mice at 4 and 12 weeks of age (Fig. 5c; *P* = 0.001 both, F(1, 5) = 14.6, *P* = 0.001); however, no significant differences were observed at 20 weeks of age (Fig. 5c; *P* = 0.30). Furthermore, pyruvate and lactate levels were significantly higher in the cochlea of *Cdk5rap1-*KO mice than those in the CNT mice at 20 weeks (Fig. 5d, e; pyruvate, *P* = 0.01, F(1, 5) = 12.6, *P* = 0.04; lactate, *P* = 0.003, F(1, 5) = 24.6, *P* = 0.03).

### Endocochlear potentials are decreased in the KO mice

**Fig. 6:**
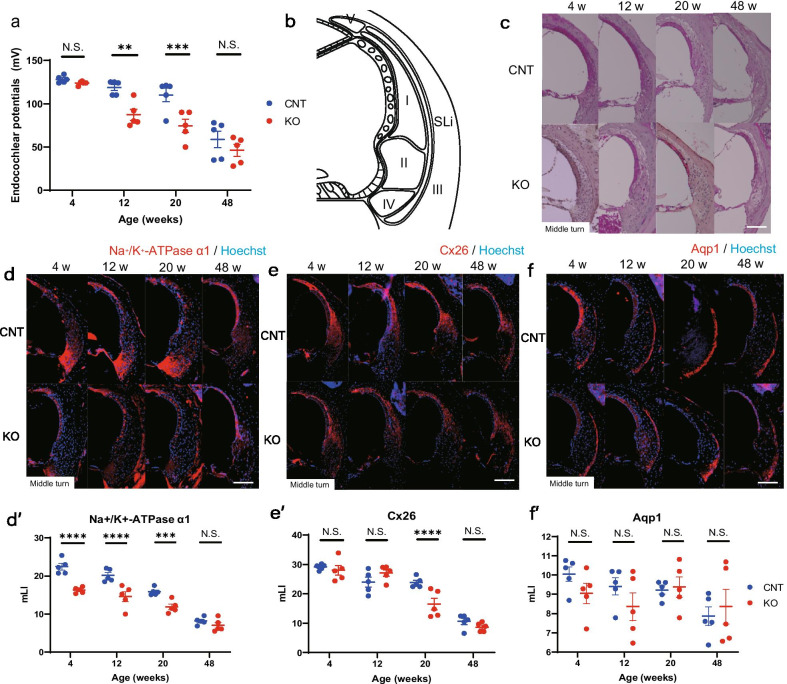
Endocochlear potential and immunohistological analysis of spiral ligament (SLi). **a** Endocochlear potentials for *Cdk5rap1*-knockout (KO) and littermate control (CNT) mice at 4, 12, 20, or 48 weeks of age. **b** Schematic representation of the SLi illustrating the five types of fibrocytes. **c** H&E staining of the SV and SLi of CNT and KO mice at different ages. **d** Immunohistochemistry of SLi of CNT and KO mice at different ages. Na^+^/K^+^-ATPase α1 (red) for types II, IV, and V. **e** Immunohistochemistry of SLi of CNT and KO mice at different ages. Connexin 26 (Cx26; red) for type I. **f** Immunohistochemistry of SLi of CNT and KO mice at different ages. Aquaporin 1 (Aqp1; red) for type III. Scale bar = 100 µm. **d’** The modified labeling index (mLI) of Na^+^/K^+^-ATPase α1 observed in CNT and KO mice at different ages. **e’** The mLI of Cx26 observed in CNT and KO mice at different ages. **f’** The mLI of Aqp1 observed in CNT and KO mice at different ages

Next, we assessed the endocochlear potentials (EPs) because we observed fibrocyte degeneration in the SLi of *Cdk5rap1-*KO mice at young ages. At 12 and 20 weeks of age, but not at 4 and 48 weeks, EPs were significantly lower in the KO mice than those in the CNT mice (Fig. 6a; 4 weeks, *P* = 0.33; 12 weeks, *P* = 0.001; 20 weeks, *P* < 0.001; 48 weeks, *P* = 0.16, F(3, 32) = 48.1, *P* < 0.001). While histological analysis revealed that the SV thickness did not differ between the KO and CNT mice at any age (Fig. 6b, c), immunohistochemical analysis indicated that the expression of Na^+^/K^+^-ATPase α1 in the SLi of KO mice was significantly reduced from 4 weeks of age (Fig. 6b, d, d’, 4 weeks, *P* < 0.001; 12 weeks, *P* < 0.001; 20 weeks, *P* = 0.003; 48 weeks, *P* = 0.99, F(3, 32) = 4.40, *P* = 0.01). Additionally, in KO mice, connexin-26 (Cx26) expression was significantly reduced from 20 weeks of age (Fig. 6b, e, e’, 4 weeks, *P* = 0.04; 12 weeks, *P* = 0.16; 20 weeks, *P* = 0.001; 48 weeks, *P* = 0.32, F(3, 32) = 5.48, *P* = 0.004) despite the lack of a significant reduction in aquaporin-1 levels (Fig. 6b, f, f’, 4 weeks, *P* = 0.84; 12 weeks, *P* = 0.77; 20 weeks, *P* = 0.99; 48 weeks, *P* = 0.99, F(3, 32) = 1.01, *P* = 0.40).

### Degenerating mitochondria are present in the SLi fibrocytes of the KO mice

**Fig. 7:**
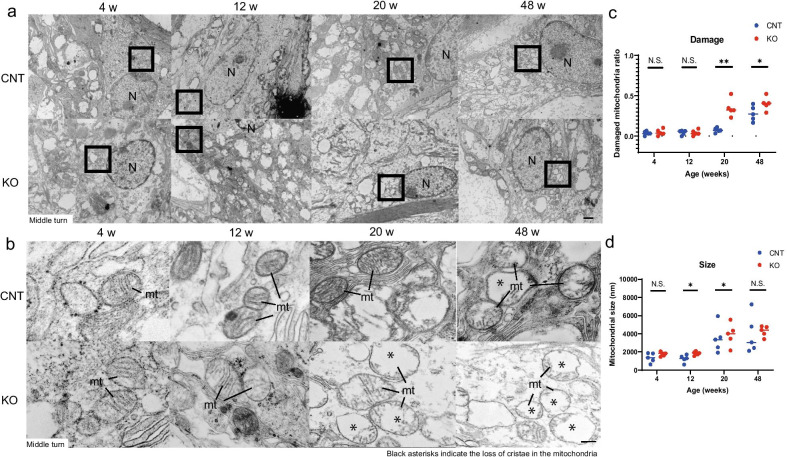
Disruption of type II and IV fibrocytic mitochondria in the spiral ligament of *Cdk5rap1-*KO mice.** a** Transmission electron microscopic images of type II and IV fibrocytes in SLi from the cochlear middle turn. *N* nuclei. Scale bar = 1 µm. **b** Magnified images of the square boxes in A. The structure of cristae in the mitochondria of *Cdk5rap1-*knockout (KO) or littermate control (CNT) mice at different ages. Asterisks indicate the loss of mitochondrial cristae. *mt* mitochondria. Scale bars = 100 nm. **c** Ratio of damaged mitochondria in KO and CNT mice. **d** Mitochondrial size in KO and CNT mice at different ages

We performed transmission electron microscopy (TEM) to investigate fibrocyte degeneration in SLi and EP reduction at early ages in the KO mice. TEM revealed that mitochondria from type II and IV fibrocytes from the SLi of KO mice started losing the cristae of the inner membrane from 20 weeks of age, which was earlier than the loss observed in the CNT mice (Fig. 7a, b). Moreover, the mitochondria of KO mice were significantly more damaged than those of CNT mice at 20 and 48 weeks of age (Fig. 7c; *P* = 0.001 and *P* = 0.03, respectively, F(1, 5) = 15.4, *P* = 0.01). Furthermore, the mitochondria of KO mice were significantly larger than those of CNT mice at 12 and 20 weeks of age (Fig. 7d; *P* = 0.01 and *P* = 0.01, respectively, F(1, 5) = 25.7, *P* = 0.003).

### Early apoptosis, ROS generation, and senescence in Cdk5rap1-KO cells

**Fig. 8:**
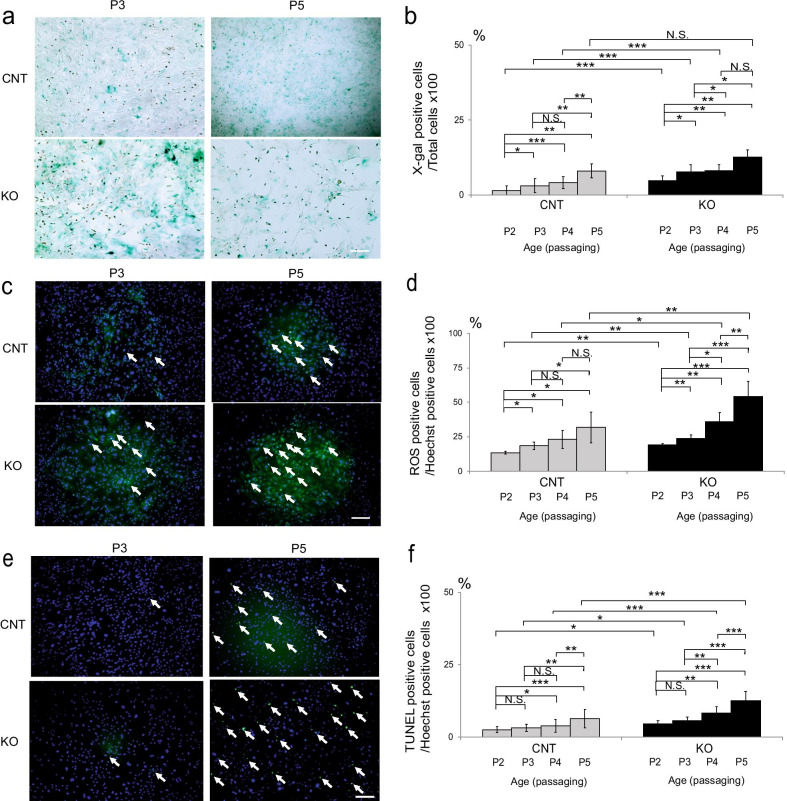
Early apoptosis, ROS generation, and senescence in *Cdk5rap1*-KO cells. **a, b** Proportion of senescent cells (blue) in *Cdk5rap1*-knockout (KO) and littermate control (CNT) cell passages (P2–5) (*n* = 5 for each genotype) **(c–f)** Proportion of **c, d** ROS-positive (green fluorescence, white arrows) and **e**, **f** TUNEL-positive (green fluorescence, white arrows) cells in each subculture of KO and CNT cells. (ROS: *n* = 5 for each genotype, TUNEL: *n* = 3 for each genotype); Scale bar = 100 µm

To verify whether in *Cdk5rap1-*KO mice aging-associated apoptosis occurred due to oxidative stress and cellular senescence, we investigated the embryonic fibroblasts of these mice. SA-β-gal activity was measured to assess cellular senescence [24, 25]. While the ratio of senescent to non-senescent cells increased significantly with each passage in both KO and CNT mice, the KO mice exhibited a greater increase (Fig. 8a, b; P2, *P* < 0.001; P3, *P* = 0.003; P4, *P* = 0.004; F(1, 5) = 47.9, *P* < 0.001). However, this effect was not observed in the P5 cells (Fig. 8a, b; *P* = 0.057). Lastly, the increase in the proportion of ROS-positive and TUNEL-positive cells was higher in the KO cells than that in the CNT cells (Fig. 8c–f; ROS: P2, *P* = 0.003; P3, *P* = 0.004; P4, *P* = 0.012; P5, *P* = 0.009; F(1, 5) = 29.4, *P* = 0.003, TUNEL: P2, *P* = 0.014; P3, *P* = 0.013; P4, *P* < 0.001; P5, *P* < 0.001; F(1, 5) = 20.8, *P* = 0.005).

## Discussion

To examine the effect of defective mitochondrial protein translation due to deficient ms^2^ modification of the mt-tRNAs on aging and AHL, we used *Cdk5rap1*-KO cells and mice as in vitro and in vivo models, respectively. In young, 4-week-old KO mice, auditory functions including ABR and DPOAE were comparable to those of the CNT mice despite an observable decrease in mitochondrial respiration; increased oxidative stress (cell-culture results); senescence of SGCs and marginal cells in the SV; degeneration of type I, II, and IV fibrocytes in the SLi; decreased expression of Na^+^/K^+^-ATPase α1 (in type II and IV fibrocytes) in the SLi; and a slight decrease of EPs. Conversely, at older ages, the *Cdk5rap1*-KO embryonic fibroblasts exhibited increased senescence, high oxidative stress, and apoptosis. Meanwhile, the KO mice exhibited hearing loss and reduced EPs compared to the those of CNT mice at 12 weeks of age. The KO mice also showed a loss of HCs and SGCs, downregulation of Cx26 (type I fibrocytes) and Na^+^/K^+^-ATPase α1 (in type II and IV fibrocytes) in the SLi, as well as disruption of the mitochondrial cristae in the type II and IV fibrocytes from the SLi at 20 weeks of age. Notably, all these changes occurred earlier in the *Cdk5rap1*-KO mice than in the CNT mice.

Mitochondrial dysfunction in the KO mice is reportedly compensated by mitonuclear protein homeostasis [22] via the induction of mitochondrial unfolded-protein response (mtUPR), which exerts a protective function [6, 22]. Moreover, a cumulative increase in expression levels of the genes involved in ROS metabolism, including the ROS-scavenger proteins, *ApoE*, *Dhcr24,* and *Srxn1*, was observed [28] under sedentary conditions [22]. Furthermore, under conditions of high-fat diet-induced metabolic stress, defective synthesis of the mitochondrial proteins was shown to cause a noticeable decrease in protein levels and the activity of complexes I and IV of the KO mice [22]. The progressive disruption of respiratory complexes exacerbates mtUPR and mitophagy; thus, compromising mitochondrial quality, function, and morphology [29]. However, the extent of mitochondrial damage is most likely overwhelming the capacity of mitophagy to maintain the mitochondrial network, leading to mitochondrial dysfunction [22, 29]. Furthermore, enhanced complex I defect was shown to be associated with a modest increase in oxidative stress, which triggers mtUPR and cytotoxicity [29]. Our results showed that auditory and cochlear functions in the *Cdk5rap1*-KO mice were maintained compared to that in the CNT under sedentary conditions (young), but not under stressed conditions (old). These results indicate that in this study aging was equivalent to stress.

In our study, the KO mice exhibited an early decrease in cochlear function due to the SLi fibrocyte degeneration, low expression of Na^+^/K^+^-ATPase α1 in the SLi, and senescence of SV preceding loss of HCs as well as SGCs. Ionic homeostasis in the endolymph, which is the extracellular fluid of the cochlear duct, is crucial for sound transduction and hearing [30]. During this process, SLi fibrocytes and SV transport of K^+^ ions, which are shuttled from the OC across the HCs back to the SLi and SV via transduction, and pumped back into the scala media [30, 31]. Chronic EP changes caused by changes in constraints on ion content imposed by the standing potentials can alter the ionic composition of the endolymph [30]. Such putative abnormalities in the ionic composition of the local endolymph might lead to HC degeneration/secondary SGC degeneration, thereby causing the changes observed in lateral-wall dysfunction [30, 32]. Our results suggest that the low Na–K-ATPase expression could compensate for low EPs by transporting ions via the fibrocytes in SV and spiral ligament of the 4-week-old KO mice compared to that of the 20-week-old CNT mice. Thus, we hypothesized that fibrocytes from the spiral ligament compensate for the low expression of Na–K-ATPase in the younger *Cdk5rap1*-KO mice. Cochlear histopathology in age-graded mice and humans indicates that AHL might result from the loss of HCs and SGCs [33]. Loss of HCs increases with age, while the loss of SGCs is consistent with the degeneration that occurs following the HC loss. In addition to these changes in HCs and their innervation via SGCs, widespread degeneration of fibrocytes from the SLi has also been reported [2, 30]. Thus, the cell loss observed in the SLi of *Cdk5rap1*-KO mice and the EP drop at a younger age preceded the loss of HCs and SGCs; thus, suggesting that the fibrocyte pathology leading to sensory-cell degeneration might cause early AHL, as such tendencies were observed in the CNT mice at older ages. Interestingly, we did not find any changes in the SV thickness with aging despite its importance for AHL pathogenesis [30, 34]. Further research investigating the SV of the KO mice is necessary for understanding the cochlear function and the aging-related modifications of mt-tRNAs.

To verify whether aging-associated stress decreased the mitochondrial oxidative metabolite levels due to deficiency of ms^2^ modification in mt-tRNAs, we analyzed the KO and CNT cochlear metabolomes at different time points. Notably, the cochlear levels of TCA cycle metabolites differed between the 12-week-old KO and age-matched CNT mice, indicating a generalized mitochondrial dysfunction consistent with decreased fumarate levels, and increased pyruvate as well as lactate levels [26, 27]. Consistent with our results and previously reported findings [6, 22, 28–30], auditory cells (HCs/SGCs) were preserved through the modification of mitonuclear protein homeostasis under sedentary conditions (young), while being disrupted following mitochondrial damage and cytotoxicity under stress conditions (old). Moreover, to investigate the SLi morphology during the early stages of AHL, we performed TEM. TEM revealed mitochondrial disruption (i.e., loss of cristae and ballooning of mitochondria) in SLi fibrocytes from the KO mice during early AHL stages, supporting the hypothesis that mitochondrial dysfunction—depending on its degree—may not immediately result in a pathological phenotype under sedentary conditions. These results suggested that the deficiency of mt-tRNA modifications cause oxidative stress in the entire cochlea, and mitochondrial dysfunction in SLi, which induce cytotoxicity in fibrocytes, resulting in EP reduction early during AHL development.

Therefore, based on our findings, we postulate that exhaustion of the ion-transport system in and around SV (i.e., fibrocytes from SLi) caused by compromised mitochondrial function due to deficiency of ms^2^ modifications in mt-tRNAs, underlies the AHL observed in the *Cdk5rap1*-KO mice. Hearing is compensated by the remodeling of mitonuclear protein homeostasis and the ion-transport system, which may inhibit oxidative stress in the younger animals [6, 28]. With age, progressive disruption of the respiratory complexes compromises mitochondrial quality control in the SLi fibrocytes, causing SV and SLi cellular senescence as well as a chronic reduction in EPs. The resulting abnormalities in the local endolymphatic ion composition might promote HC degeneration and the loss of secondary SGCs. Previous studies have shown that dysregulated tRNA modifications precede aging in humans [9, 10]. Therefore, in humans, AHL might be caused by cochlear energy loss due to decreased post-transcriptional modifications in mt-tRNAs.

A primary limitation of our in vivo study is that using the murine C57BL/6 model may not accurately represent AHL, although the etiology and pathology of hearing loss are similar to those of AHL. The C57BL/6 strain has a critical mutation in the cadherin 23 (*Cdh23)* gene [35], the expression of which is critical for maintaining the organized, staircase-like structure of the stereocilia hair bundle due to its role in the interciliary links [36, 37]. The AHL phenotype linked to this mutation has been shown to be initially induced by the disruption of the stereociliary bundle, followed by hair-cell loss, spiral-ganglion loss, and dysfunction of potassium ion recycling via cochlear fibrocytes [34, 38]. Thus, C57BL/6 mice are prone to AHL due to this *Cdh23* mutation [35]. Ideally, this research should have been conducted using other strains, such as CBA or BALB/c, which have been backcrossed with a strain without any inherent hearing problem. Additionally, our mouse model showed initiation of SLi degeneration at 4 weeks of age. Investigation of the cochlear morphology preceding the onset of SLi degeneration was not possible in the present study as we only analyzed the adult mice; therefore, further research may be warranted to consider this issue. Moreover, we did not further investigate SV or SGCs despite detecting their senescence, which is important for the histopathological assessment of AHL [30, 38–40]. Furthermore, we did not find any age-related changes in SV thickness despite early aging and increased apoptosis in *Cdk5rap1*-KO MEFs and the importance of SV thickness for AHL pathogenesis [30, 34]. Thus, further studies investigating the SV and SGCs are required to better understand their roles in AHL. While we defined senescent cells as the cells expressing SA-β-gal staining in vivo and in vitro, we did not reveal whether the labeled cells were undergoing degeneration or remained in this state for a long period of time. Furthermore, we observed TEM artifacts in our study. Microstructural TEM images frequently contain artifacts such as vacuolization caused by insufficient fixation. Thus, further TEM studies are required to study the cochlear mitochondrial structure during aging.

We provided evidence of mitochondrial dysfunction caused by deficient CDK5RAP1, which catalyzes the ms^2^ modifications of mt-tRNAs. Such mitochondrial dysfunction leads to cell senescence and AHL. Our results suggest that the accumulation of dysfunctional mitochondria may lead to AHL progression. Our findings help understand the mechanisms underlying AHL and offer valuable insights into the association between aberrant tRNA modification-induced mitochondrial dysfunction and hearing loss. Elucidation of the mechanisms underlying AHL would guide future clinical interventions aimed at mitigating the AHL outcomes.

## Materials and methods

### Animals

Sixty 4-week-old female *Cdk5rap1-* heterozygous C57BL/6 J mice were provided by Prof. Kazuhito Tomizawa (Kumamoto university). KO mice were generated by crossing the transgenic mice exhibiting the exons 5 and 6 of *Cdk5rap1* floxed with the *Lox*P sequence, with transgenic mice expressing Cre recombinase under the control of the CAG promoter. Mice were backcrossed with C57BL6/J mice for at least seven generations to eliminate the Cre transgene and control the genetic background. KO mice and the control littermates (4 weeks old) were used in experiments unless otherwise specified. Detailed information on genotyping is available upon request. Groups of five mice each were housed together in a cage in a temperature-controlled room maintained at ~ 25 °C and ~ 50% humidity. All animals had ad libitum access to a standard commercial pellet diet and water. Animals were randomly assigned to experimental groups. All mice were euthanized by cervical dislocation.

### Tissue processing

Inner ears of the *Cdk5rap1-*KO and CNT mice were dissected at 4, 12, 20, or 48 weeks after birth. Cochleae were extracted and fixed for 12 h at 4 °C in 4% paraformaldehyde prepared in phosphate-buffered saline (PBS) and then decalcified for three days at 25 °C in 0.5 mol/L EDTA (Wako, Osaka, Japan). Next, tissues were embedded in optimal-cutting-temperature medium (Sakura Finetek Japan, Tokyo, Japan), frozen, and serially cryosectioned at 12-µm thickness for subsequent examination.

### H&E staining

Histological staining was performed using H&E to assess the gross morphology (*n* = 5 for each genotype). Images were obtained using a BZ-9000 fluorescence microscope (Keyence, Osaka, Japan).

### Immunohistochemistry

For antigen detection, the following primary antibodies and dilutions were used: anti-Na^+^/K^+^-ATPase α1 (1:200; cat. No. NB300-146; Novus, Centennial, CO, USA), anti-aquaporin-1 (Aqp1, 1:200; cat. No. AQP-001; Alomone Labs, Jerusalem, Israel), and anti-Cx26 (1:200; cat. No. 710500; Life Technologies, Carlsbad, CA, USA). The following fluorophore-conjugated secondary antibodies were used: Alexa Flour 594 goat anti-mouse (1:500; cat. No. A-11032, Thermo Fisher Scientific, Waltham, MA, USA) and Alexa Flour 594-conjugated goat anti-rabbit (1:500; cat. No. A32740; Thermo Fisher Scientific). After blocking in 10% goat serum in PBS for 10 min, the sections were incubated overnight with primary antibodies at 4 °C and subsequently probed with the secondary antibodies at 25 °C for 1 h. Samples were then counterstained using Hoechst 33,258 dye (Molecular Probes, Eugene, OR, USA) for 30 s for nuclear staining, and imaged using a BZ-9000 fluorescence microscope (*n* = 5 for each genotype).

### Modified labeling index (mLI)

We used mLI to evaluate the immunostaining levels in the cryosectioned samples as described previously [41, 42]. Briefly, images were captured at 1360 × 1024 pixels under the same photographic exposure conditions. Tissue-free areas outside the proper cochlear tissue were selected using the “Magic Wand” tool of Adobe Photoshop (Adobe Systems, San Jose, CA, USA) after adjusting the tolerance level of the tool to enable the selection of these areas. The selected tissue-free areas were used for background adjustment. Each stained area was determined in a similar manner. Images were converted to the 16-bit grayscale format. The optical densities of background areas and each stained area were assessed using the “Histogram Tool” of Adobe Photoshop. The mean staining intensity and number of pixels in each area were determined. Final staining intensity was calculated as the difference between the mean staining intensity and mean background intensity. Staining ratios were estimated as the ratio of the number of pixels in each stained area to that in the entire image. The mLI measurements were performed in a blinded manner (*n* = 5 for each genotype).

### Counting the SGCs

SGCs were assessed using immunohistochemistry. After blocking with 10% goat serum in PBS, sections were incubated with a mouse polyclonal anti-beta III tubulin (Tuj1) antibody (1:200; cat. No. PRB-435P; Covance, Princeton, NJ, USA) for 1 h. The sections were washed thrice in PBS and incubated with Alexa Fluor 555-conjugated goat anti-mouse antibody (1:500; Thermo Fisher Scientific) at 25 °C for 1 h. Hoechst 33,258 dye (1:1000) was used for 30 s for nuclear staining. Sections were imaged using a BZ-9000 fluorescence microscope. Three randomly selected images from each cochlear turn were captured for each group. We enumerated the cells positive for both Tuj-1 and Hoechst from the Rosenthal’s canal as SGCs at the basal, middle, and apical turns in three randomly selected sections per cochlea and marked counts on the images to avoid double-counting. Cells were counted using the ImageJ software (Ver. 1.52 h, NIH, Bethesda, MD, USA) as previously described [43, 44]. The accuracy of the analyses was verified by an independent second researcher (*n* = 5 for each genotype).

### Hair-cell count

To assess the cochlear surface morphology, the *Cdk5rap1-*KO and CNT mice were fixed using 4% paraformaldehyde by cardiac perfusion under deep anesthesia induced by intraperitoneal injection of 4 mg/kg xylazine (Bayer, Shawnee Mission, KS, USA) and 120 mg/kg ketamine-HCl (Daiichi Sankyo, Tokyo, Japan) in 0.9% NaCl. Following fixation, the bony capsule and lateral wall of the cochlea were removed. Samples were incubated with Texas Red-X phalloidin (1:100; Molecular Probes) for 30 min to stain F-actin. The cochlear surface morphology was imaged using a BZ-9000 fluorescence microscope. Five randomly selected surface images of the OC were captured at every turn of the cochlea at 40 × magnification for each group. The nusmber of IHCs and OHCs in a 140-μm basal segment of the basilar membrane was counted. Only HCs with an intact stereociliary bundle and a cuticular plate were counted per cochlear turn as previously described [32, 44, 45]. A second researcher verified the accuracy of the results (*n* = 5 for each genotype).

### Quantitative assessment of senescence-associated (SA)-βgal

Senescent cells were detected using an SA-βgal kit (Cosmo Bio Co., Ltd., Tokyo, Japan). Images were obtained using a BZ-9000 fluorescence microscope and quantified using CellProfiler (http://www.cellprofiler.org/). Briefly, we retrieved grayscale images from the UnmixColors module and scored the blue signals in the entire cochlea and/or parts of it. Investigators were blinded to the groups during the experiments and analyses (*n* = 5 for each genotype).

### TEM

Each mouse was briefly perfused with 0.9% NaCl through the ascending aorta followed by fixation in a 20 mL solution of 2.5% glutaraldehyde and 2 mM CaCl_2_ prepared in 0.1 M sodium cacodylate (pH 7.4). The inner ear was dissected. Samples were prefixed using a mix of 2% paraformaldehyde and 2% glutaraldehyde prepared in PBS (pH 7.4) at 25 °C. Post-fixation was performed on ice for 30 min using 2% osmium tetroxide. Subsequently, samples were stained with 1.5% uranyl acetate for 1 h at 4 °C and then dehydrated in an ascending ethanol gradient (50, 70, 90, and 100%) as well as 100% propylene oxide, embedded in Epon-Araldite, and polymerized at 60 °C for 48 h. Samples were sliced at 60–65 nm using an ultramicrotome (UC7i, Leica, Munich, Germany) and stained with 1.5% uranyl acetate and fresh Reynolds’ lead citrate at 25 °C. Specimens were examined using a transmission electron microscope (HT7700, Hitachi, Tokyo, Japan; *n* = 5 for each genotype). Damaged (characterized by structural abnormalities) and normal mitochondria in each image were enumerated, and the ratio of damaged mitochondria to normal mitochondria was determined. Furthermore, the size of oblong mitochondria in each image was estimated using ImageJ (*n* = 5 for each genotype).

### Auditory brainstem response (ABR)

Auditory thresholds were measured using an ABR System 3 (Tucker-Davis Technologies, Alachua, FL, USA). Anesthesia was induced via intraperitoneal injection of 4 mg/kg xylazine and 120 mg/kg ketamine-HCl in 0.9% NaCl. The electrodes were placed beneath the pinna of the treated ear and at the vertex right below the surface of the skin, while a ground electrode was placed under the contralateral ear. An average of 512 sweeps was calculated for 4, 8, 12, 20, or 32 kHz. Stimulus levels near the threshold were varied in 10-dB steps. The threshold was defined as the lowest level at which the ABR waves could be visually detected [41, 44, 45] (*n* = 5 for each genotype).

### Otoacoustic emissions

For measuring DPOAE, animals were anesthetized as previously described and pinnae were removed. An ER10B + probe microphone–speaker system with two speaker ports (Etymotic Research, Inc., Elk Grove Village, IL, USA) was fitted tightly into the ear canal and linked to two closed-field MF-1 speakers (Tucker-Davis Technologies). Two primary tones were generated (1-s duration with 20 ms rise–fall cosine ramp; f 2/f 1 = 1.22, f 2 varied from 4 to 20 kHz) and routed separately to the two MF-1 magnetic speakers at SPL1 = 75 dB and SPL2 = 65 dB. The SPL was calibrated in a 0.1-mL coupler using a Brüel and Kjær 1/4″ pressure field microphone (model 4938), which has a flat frequency response from 4 Hz to 70 kHz. The calibration was conducted for primary tones and all DPOAE components. The DPOAE response from the ER10B + microphone was amplified by 20 dB and digitized at 150 kHz using a NI USB-6216 signal processor and analog-to-digital converter (National Instruments, Austin, TX, USA). Data were acquired and analyzed using customized software written using Labview 2015 (National Instruments). Recordings were repeated 10 times at 20-s intervals and averaged as a function of time. The noise was estimated by averaging three adjacent frequency bins above and below the DPOAE frequency [44] (*n* = 5 for each genotype).

### Endocochlear potentials

EP recordings were performed as previously described [44, 45], at each age stage, under general anesthesia. Briefly, the cochlea was first ventrally exposed. The bone over the SLi was thinned, and a small opening was generated using Bonn microprobes (#10,030-13, F.S.T, Vancouver, BC, Canada) to access the endolymphatic compartment (scala media) of the basal turn. A heat-pulled micropipette electrode filled with 150 mM KCl was inserted into this compartment until a stable potential, defined as the point at which the potentials no longer depend on electrode depth, was recorded. The signal was amplified using an MEZ-7200 amplifier (Nihon Koden, Tokyo, Japan) and the direct current potentials were recorded using a USB-6216 analog-to-digital converter (National Instruments; *n* = 5 for each genotype).

### Metabolomic analysis

Cochleae of 4-, 12-, 20-, or 48-week-old *Cdk5rap1-*KO or CNT mice were dissected, washed in PBS, and frozen at -80 °C until metabolite extraction. Metabolites were extracted using methanol containing HMT (Human Metabolome Technologies, Yamagata, Japan) as the internal standard solution at 25 °C. Metabolites were detected using gas chromatography coupled with mass spectrometry (GC–MS; Shimadzu Co., Tokyo, Japan) [46]. Peaks were detected using multivariate data-analysis software (Travers MS, Reifycs Inc., Tokyo, Japan) in a three-step manner: (1) mass values were detected within each spectrum; (2) a chromatogram spanning a certain time range was constructed for each mass value; and (3) deconvolution algorithms were applied to each chromatogram to identify the chromatographic peaks. The average peak height for each metabolite was analyzed (*n* = 3 for each genotype).

### Cell culture

*Cdk5rap1-*KO or CNT mouse embryonic fibroblasts, provided by Prof. Kazuhito Tomizawa (Kumamoto University) [22] were cultured in 24-well plates (0.2 × 10^6^ cells/well) in a high-glucose OptiMEM medium (Thermo Fisher Scientific) supplemented with 10% fetal bovine serum (Thermo Fisher Scientific) and 1% penicillin/streptomycin solution (Thermo Fisher Scientific) at 37 °C and 5% CO_2_. After reaching confluence, cells were trypsinized and subcultured for a maximum of five times to avoid subculturing-induced cellular senescence [47].

### Cell-culture assays

Between passages 2 (P2) and 5 (P5), *Cdk5rap1-*KO or CNT cells were washed with PBS and fixed with 4% paraformaldehyde. Senescent cells were detected using an SA-βgal kit (Cosmo Bio Co., Ltd), ROS were detected using the CellROX™ Green Reagent (Thermo Fisher Scientific), while apoptotic cells were detected using the TUNEL staining (Sigma-Aldrich, St. Louis, MO, USA) as per manufacturers’ instructions. During ROS detection and TUNEL staining, Hoechst 33,258 dye was used to counterstain the nuclei for cell counting. For quantitative analyses, the total cell number and the number of SA-β-gal-positive cells were determined in three randomly selected microscopic fields (*n* = 5 for each genotype). ROS-, TUNEL-, or Hoechst-positive cells were counted in a similar manner. Cells were enumerated by a researcher blinded to the murine genotype and age (ROS: *n* = 5 for each genotype, TUNEL: *n* = 3 for each genotype).

### Experimental design and statistical analyses

Eighty female *Cdk5rap1*-KO mice and eighty CNT female littermates were used following genotyping. Data were analyzed using two-way ANOVA and post hoc Tukey tests. Statistical analyses were performed using GraphPad Prism version 8.0.0 for Windows (GraphPad Software, San Diego, CA, USA, www.graphpad.com). Multivariate analyses including heatmap generation, principal-component analysis, and enrichment analyses were performed using MetaboAnalyst (https://www.metaboanalyst.ca/) [48]. Data correction was performed using familywise error rate via Bonferroni analysis. The data met the assumptions of the statistical tests utilized. The statistical power and the sample size were determined before and after data collection using PS: Power and Sample Size Calculation, Ver. 3.1.6 (Department of Biostatistics, Vanderbilt University, Nashville, TN, USA) [49]. Data are expressed as mean ± standard error (SE). *P*-values < 0.05 were considered statistically significant.

## Supplementary Information

**Additional file 1.** ABR wave form of 12 kHz, 90 dB SPL in Cdk5rap1-KO and CNT mice at different ages.

**Additional file 2.** Magnified images of H&E staining of the middle cochlear turn in Cdk5rap1-KO and CNT mice at different ages. (a) OC, (b) SGCs, (c) SV and SLi. Scale bar = 100 µm.

**Additional file 3.** Magnified images of SA-β-gal assay of middle cochlear turn in Cdk5rap1-KO and CNT mice at different ages. (a) OC, (b) SGCs, (c) SV and SLi. Scale bar = 100 µm.

**Additional file 4. **Lipofuscin detection using H&E staining of the middle cochlear turn in Cdk5rap1-KO and CNT mice at different ages. (a) OC, (b) SGCs, (c) SV and SLi. Black arrows indicate lipofuscin. Scale bar = 100 µm.

**Additional file 5. **Heatmap analysis of the metabolome results.

**Additional file 6. **Metabolome principal-component analysis.

**Additional file 7.** Metabolome enrichment analysis via metabolome analysis.

## Data Availability

The datasets used and/or analyzed in the present study are available from the corresponding author on reasonable request.

## Abbreviations

ABR: Auditory brainstem response
DPOAE: Distortion-product otoacoustic emissions
AHL: Age-related hearing loss
CDK5RAP1: Cdk5 regulatory subunit-associated protein 1
CNT: Control
EP: Endolymphatic potential
HC: Hair cell
IHC: Inner hair cell
KO: Knockout
mtDNA: Mitochondrial DNA
OHC: Outer hair cell
ROS: Reactive oxygen species
SE: Standard error
SGC: Spiral-ganglion cell
SLi: Spiral ligament
SV: Stria vascularis
TCA: Tricarboxylic acid
TEM: Transmission electron microscopy
tRNA: Transfer RNA
Tuj1: Beta III tubulin
