# Supplementary figures and images for: Cdk5 regulatory subunit-associated protein 1 knockout mice show hearing loss phenotypically similar to age-related hearing loss
Source: Mol Brain. 2021 May 17;14:82. doi: 10.1186/s13041-021-00791-w (PMC8130336; doi:10.1186/s13041-021-00791-w)

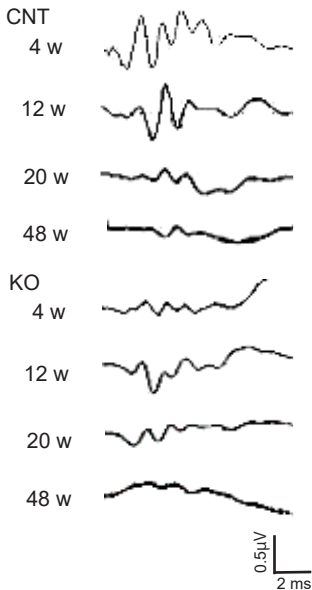

### **Additional file 1.**

ABR wave form (12 kHz, 90 dB SPL)

Supplement: Supplementary file 1 — Additional file 1. ABR wave form of 12 kHz, 90 dB SPL in Cdk5rap1-KO and CNT mice at different ages. [file 13041_2021_791_MOESM1_ESM.pdf]

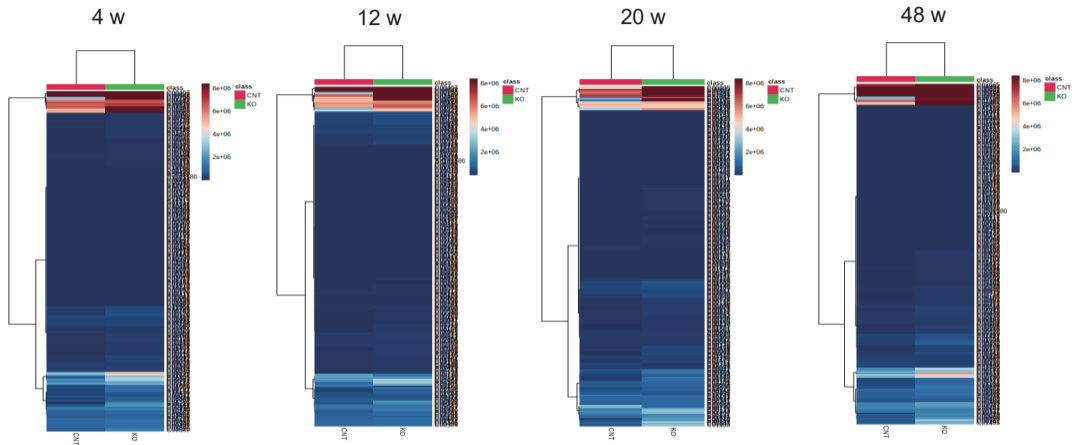

**Additional file 5.** Changes in mitochondrial metabolite species with age in heatmap analysis.

Supplement: Supplementary file 5 — Additional file 5. Heatmap analysis of the metabolome results. [file 13041_2021_791_MOESM5_ESM.pdf]

4 w

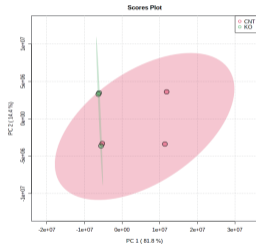

12 w

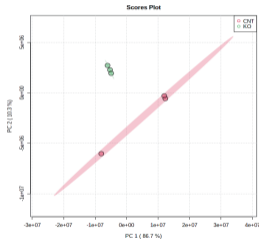

20 w

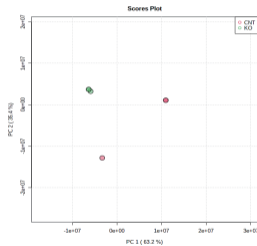

48 w

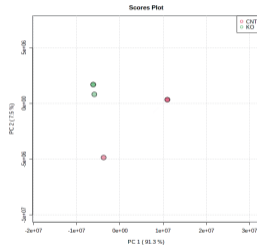

**Additional file 6.** Changes in mitochondrial metabolite species with age in PCA analysis.

Supplement: Supplementary file 6 — Additional file 6. Metabolome principal-component analysis. [file 13041_2021_791_MOESM6_ESM.pdf]
