# Supplementary material for: Cdk5 regulatory subunit-associated protein 1 knockout mice show hearing loss phenotypically similar to age-related hearing loss
Source: Mol Brain. 2021 May 17;14:82. doi: 10.1186/s13041-021-00791-w (PMC8130336; doi:10.1186/s13041-021-00791-w)
Supplement: Supplementary file 4 — Additional file 4. Lipofuscin detection using H&E staining of the middle cochlear turn in Cdk5rap1-KO and CNT mice at different ages. (a) OC, (b) SGCs, (c) SV and SLi. Black arrows indicate lipofuscin. Scale bar = 100 µm. [file 13041_2021_791_MOESM4_ESM.pdf]

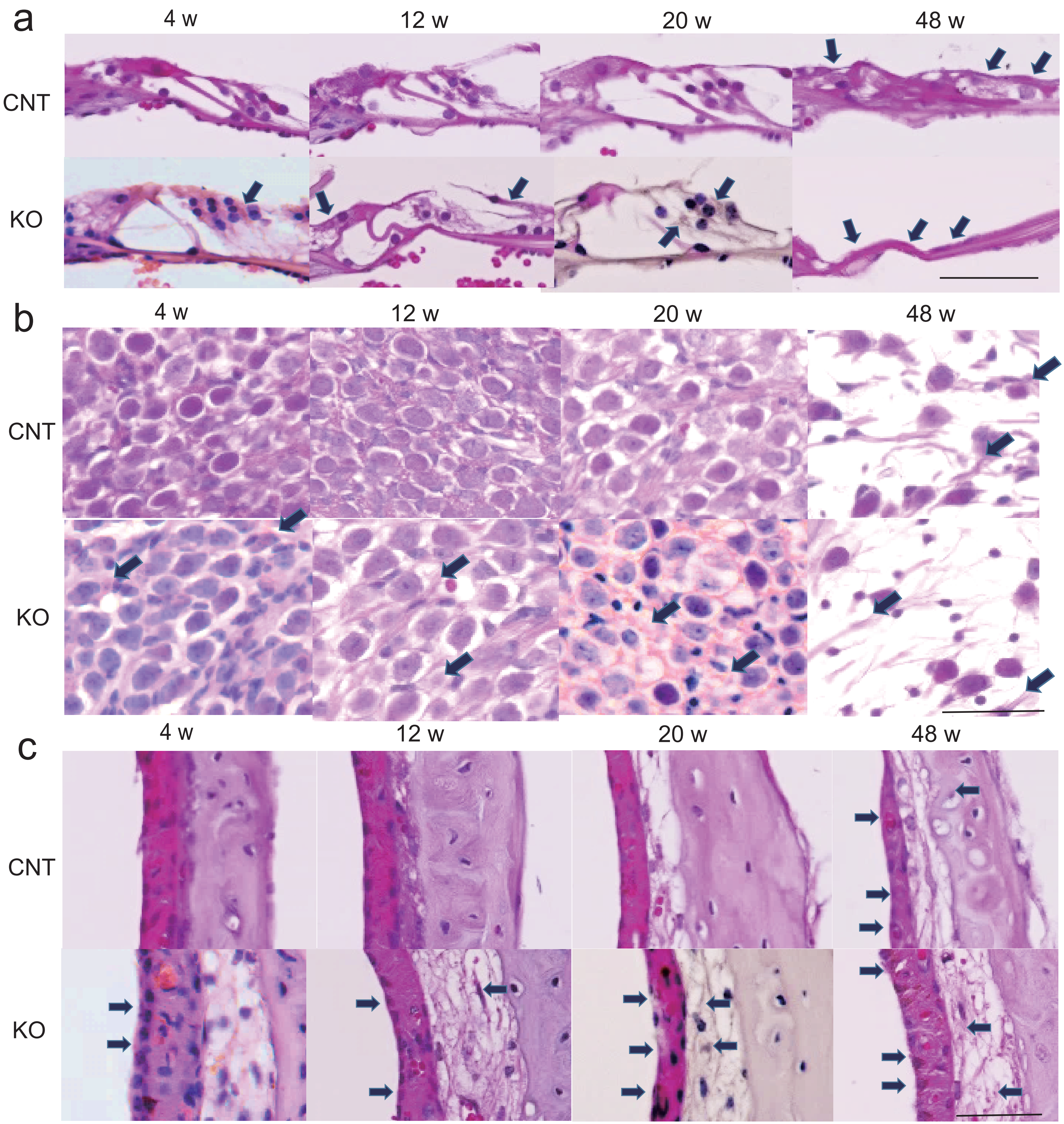

Additional file 4. Lipofuscin detection via H&E staining of middle turn in cochlea in KO and CNT mice at different ages.
