# Supplementary material for: Cdk5 regulatory subunit-associated protein 1 knockout mice show hearing loss phenotypically similar to age-related hearing loss
Source: Mol Brain. 2021 May 17;14:82. doi: 10.1186/s13041-021-00791-w (PMC8130336; doi:10.1186/s13041-021-00791-w)
Supplement: Supplementary file 7 — Additional file 7. Metabolome enrichment analysis via metabolome analysis. [file 13041_2021_791_MOESM7_ESM.pdf]

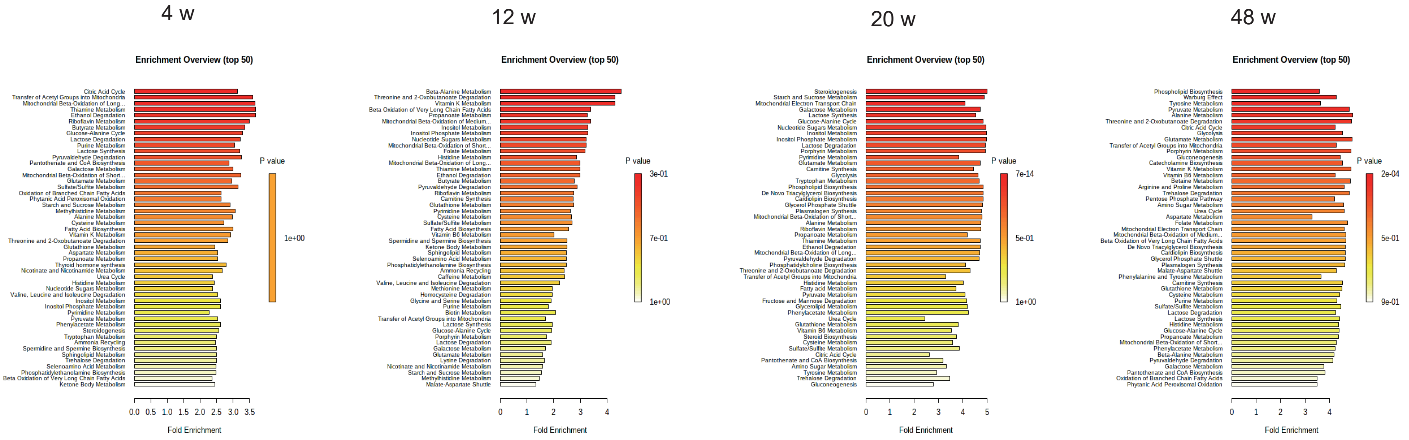

Additional file 7. Changes in mitochondrial metabolite species with age in enrichment analysis.
